# Monthly Summary

**Published:** 1883-10

**Authors:** 


### MONTHLY SUMMARY.

---

THE EFFECT OF TOBACCO ON YOUTH.—Dr. G. Decaisne has submitted to the Society of Public Medicine the results of some interesting observations concerning the effects due to the the use of tobacco among boys. Thirty-eight youths were placed in his charge, whose ages varied from nine to fifteen, and who were in the habit of smoking, though the abuse of tobacco varied in each case. The effects of course also varied, but were very emphatic with twenty-seven out of the thirty-seven boys. With twenty-two patients there was a distinct disturbance of the circulation, bruit at the carotids, palpitation of the heart, deficiency of digestion, sluggishness of the intellect, and a craving, more or less pronounced, for alcoholic stimulants. In thirteen instances there was an intermittent pulse. Analysis of the blood showed in eight cases a notable falling off in the normal number of red corpuscles. Twelve boys suffered frequently from bleeding of the nose. Ten complained of agitated sleep and constant nightmare. Four boys had ulcerated mouths, and one of the children became the victim of pulmonary phthisis, a fact which Dr. Decaisne attributed to the great deterioration of the blood produced by prolonged and excessive use of tobacco. As these children were all more or less lymphatic, it was not possible to establish a comparison according to temperament; but of course the younger the child the more marked were the symptoms, and the better-fed children were those that suffered least. Eleven had smoked for six months, eight for one year, and sixteen for more than two years. Out of eleven boys who were induced to cease smoking, six were completely restored to normal health after six months, while the others continued to suffer slightly for a year. Treatment with iron and quinine gave no satisfactory result, and it seems tolerably evident that the most effective, if not the only cure, is to at once forswear the habit, which to children in any case is undoubtedly pernicious. —*Lancet.*

---

ANOTHER DEATH FROM CHLOROFORM.—The *Med. Press.* August 8. 1883, reports another case. The man, aged 46, was chloroformed for the excision of a tumor of the lip. Soon after the inhalation was commenced, the patient became livid, and the efforts to restore respiration, which quickly ceased, were unavailing. Examination of the body *post mortem* showed that all the organs were in a healthy condition, and a verdict was returned to the effect that "deceased died from sudden paralysis of the nerves caused by chloroform, which had been properly administered."

These cases teaches us to use great caution in the administration of chloroform, and we would lay it down as a cardinal rule that the inhalation should be very gradual—that is, plenty of air should be allowed to be inspired with the chloroform until the system has commenced to tolerate the toxic effects of the anæsthetic. The common practice of pouring chloroform on a towel and clapping it over the patients nose and mouth, is to be greatly deprecated.—*Med. and Surg. Reporter.*

